# Masked invader in Iran! Habitat suitability analysis for invasive raccoon (*Procyon lotor*) in the west of Guilan Province

**DOI:** 10.1002/ece3.70090

**Published:** 2024-08-06

**Authors:** Amin Hekmat, Saeid Naderi, Wahid Zamani

**Affiliations:** ^1^ Natural Resources Faculty, Department of Environment University of Guilan Rasht Iran; ^2^ Department of Environmental Sciences, Faculty of Natural Resources University of Kurdistan Sanandaj Iran

**Keywords:** Guilan Province, habitat suitability model, invasive species, MaxEnt, raccoon

## Abstract

Nowadays, in addition to the destruction and fragmentation of the world's habitats, invasive species, and damage caused by them, are one of the most important factors in the destruction of ecosystems. The raccoon *(Procyon lotor*) is a medium‐sized mammal that is placed in mid‐levels of the food web and can affect a wide range of species. Considering the damage done to local ecosystems by this invasive species, habitat assessment and determining the factors affecting its habitat suitability would be a key step in managing this species. In this study, using the MaxEnt model and examining 12 environmental parameters (elevation, slope, aspect, geological units, soil type, vegetation, land use, distance to villages, distance to main roads, distance to waterways, average temperature, and rainfall) in the west of Guilan Province, habitat suitability of this alien species was determined, and the most important factors affecting this suitability were investigated. Results showed that the validity value of the model (AUC) was estimated to be 0.852 and parameters such as distance to village (34.5%), elevation (24.2%), and land use (15.9%) are among the most important and effective factors. Also, the results showed that 0.60% of the study area has high suitability, 6.14% moderate, 24.87% low, and 68.36% unsuitable areas for raccoons. The overall result shows that despite the lack of vast favorable areas for this invasive species, an increase in the number and expansion of this species is very likely because of its omnivorous diet, high adaptability to different environments and conditions, as well as extensive niche. All of these factors cause raccoons to spread further in the region and consequently increase the risks and damages to the native ecosystem.

## Graphical abstract

Nowadays, in addition to the destruction and fragmentation of the world's habitats, invasive species and damage caused by them, are one of the most important factors in the destruction of ecosystems. The raccoon (Procyon lotor) is a medium‐sized mammal that is placed in mid‐levels of the food web and can affect a wide range of species. Considering the damage done to local ecosystems by this invasive species, habitat assessment and determining the factors affecting its habitat suitability would be a key step in managing this species. In this study, using the MaxEnt model and examining 12 environmental parameters in the west of Guilan Province, the habitat suitability of this alien species was determined and the most important factors affecting this suitability were investigated.

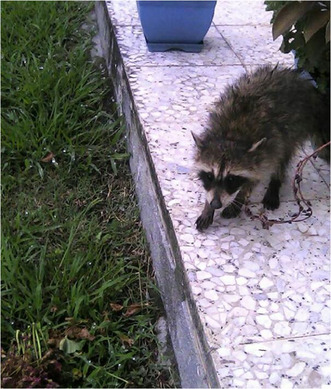

## 1 INTRODUCTION

In today's world, alongside the habitat destruction and fragmentation, introduction of alien species is one of the major threats to the environment and local communities (plant, animal, and human) (IUCN, 2004). The entrance of alien species to new habitats can cause major changes in ecosystems' structure such as hunter–prey relations, as well as competition between different species (Bartoszewicz et al., 2008). Invasive species distribute rapidly in new areas and increase in population due to favorable environmental conditions for growth and survival, lack of disease, and natural enemies (Ricciardi, 2013). Robust information about the distribution of alien and invasive species is more than necessary for biodiversity conservation, monitoring, and conservation management (Dornelas et al., 2014). Knowledge about the potential distribution of alien species is also a need for conservation managers in order to make better plans in the decision‐making process of tasks such as bio‐security (Catford et al., 2012), identification of entry points (Seebens et al., 2013) quantification of impacts posed by invasive alien species (Blackburn & Steele, 1999), and the assessment of the ecological degradation of habitats (Vandekerkhove et al., 2013). Day by day growing evidences demonstrates that not only the invasion of alien species has imposed serious threats to native biodiversity, but also threats to health and economics (Groom et al., 1997; Scalera et al., 2012; Sinkins & Otfinowski, 2012; Usher, 1988; Westman, 1990). Alien species such as raccoons have shown rapid expansion in countries like Russia, Japan, Germany, and some European countries and Canada (Duscher et al., 2021; Fischer et al., 2015; Hays & Simberloff, 2006; Hohmann et al., 2001). Given this issue and the potential dangers of this animal to human communities (Sanderson, 1987), actions toward management of this species and efforts to remove it from the area to reduce or eliminate the destructive effects of this species would be necessary.

Raccoon, *Procyon lotor* (Linnaeus, 1758) is one of the generalist mid‐sized mammals that could easily adapt to different habitat conditions (Heske & Ahlers, 2016; Hohmann & Bartussek, 2005; Johnson, 1970). With an omnivorous feeding strategy and high reproductive rate and also the absence of natural predators or enemies, it has the capability of rapid expansion in new areas (Boscherini et al., 2020; Hohmann & Bartussek, 2005). This nocturnal species starts its activities early in the evening. The home range of alien raccoons is estimated to be about 1 km^2^ in suburban areas, 10 km^2^ in wetlands, and about 60 km^2^ in forests (Bartoszewicz et al., 2008). They are highly dependent on forest habitats, especially those near water or wetlands, and make extensive use of large trees in any habitat (Gehrt, 2003). Usually, upstream habitats such as pastures and meadows are not favorable for raccoons. They use linear components such as roads or rows of fences as lanes for cruising in habitats (Fritzell, 1978; Glueck et al., 1988). According to Cunze et al. (2023), forest areas and also mixed landscapes, which include agricultural lands as well as urban areas, are the most preferred areas by raccoons. Also, other studies on this field show that forest and mixed landscapes referred to as primary raccoon habitats (Beasley et al., 2007; Heske & Ahlers, 2016; Hohmann & Bartussek, 2005) and water bodies and cities in these types of regions considered as core areas (Duscher et al., 2018; Fiderer et al., 2019).

Raccoons are common inhabitants of urban areas and their density in places where a variety of food and water resources as well as abundant shelters are available is much higher (Beasley & Rhodes Jr, 2008; Gehrt, 2004; Ikeda et al., 2004). They can pose a threat to local fauna by hunting, competing, or transmitting diseases. Also, they can have an impact on agricultural activities by hunting poultry or crops and these impacts will have consequences on the local economy. Besides the economic damages, raccoons host diseases and viruses such as raccoon roundworm, distemper virus, and rabies, some of which are dangerous to humans so they could potentially endanger the health of both human and animal communities (Beltrán‐Beck et al., 2012; Duscher et al., 2017; Gehrt, 2004; Keller et al., 2011; Lombardo et al., 2022). In areas where the number of raccoons increases too much, such as in urban parks or forests, they hunt a wide range of vertebrate preys (Hohmann & Bartussek, 2005; Robinson et al., 1995); ground‐nesting birds, such as ducks, may be more vulnerable (Garrettson & Rohwer, 2001; Greenwood et al., 1990). Species that nest in tree cavities may also be at high risk of competing for nests with raccoons. Raccoons have been introduced in different parts of the globe (Europe and some Caribbean islands) for many purposes such as presenting them in zoos, as pets, for fur industries, etc. Due to some incidents, such as escaping from zoos and fur farms, intentional or unintentional release of pet owners has become an invasive alien species in these habitats and has caused a wide range of damages from economic to human health and ecosystem damages (Duscher et al., 2021; Fischer et al., 2015). Various studies have been conducted on this animal in places where it exists as an invasive species. One of the main solutions suggested by researchers to solve the raccoon problem is complete eradication, which requires precise monitoring and detailed information on its habitat and population. If performed accurately, it is identified as a solid way to save habitats and ecosystems from raccoon invasion (Golumbia et al., 2000; Ikeda et al., 2004; Mazzamuto et al., 2020). Raccoons in Iran, as an invasive species, have been seen in Caspian areas, mostly in near water shrubs. The presence of this species was recorded by Ziaei in 1996 for the first time in Talesh city of Guilan Province. According to Ziaei (1996), this species has entered Iran from the Iran‐Azerbaijan border, which is through the border city of Astara. The presence of raccoons has been recorded in cities such as Astara, Talesh (Hashtpar), and Asalem in Guilan Province also there are the number of unconfirmed reports of its presence in Chamestan and Sari in Mazandaran Province as well (Farashi et al., 2016; Ziaei, 1996).

Previously, limited research has been conducted on this invasive species in Iran. Farashi et al. (2016) conducted research in order to show suitable habitats for raccoons around the globe using MaxEnt analysis. Their results showed that a great portion of different types of habitats are at high risk of Raccoon expansion in Iran. The results of this study also showed that precipitations and temperature are among the most important parameters for invasive Raccoon distribution and it does not prefer cold environments with average temperatures below 0°C. Farashi et al. (2011a, 2011b) also conducted a study to investigate the status of the species and factors affecting its distribution in the Lavandevil Wildlife Sanctuary using the Ecological Niche Factor Analysis (ENFA) method. The results showed that the variables of plant communities, vegetation density, and water resources are effective in determining the distribution of this species on a microscale. Throughout the year urban waste and fish were the highest and lowest portion of raccoons diet, respectively. In another study, Farashi et al. (2011a, 2011b) simulated the trend of raccoon invasion in Guilan Province using the Genetic Algorithm (GARP) technique, which showed that 36% of ecosystems in Guilan Province are at risk of this species invasion. The results of a study in order to predict raccoon expansion in Iran using MaxEnt showed that until the 2060s, raccoons could reach the western and southwestern parts of the country (Khosravifard, 2015). Also, Khosravifard et al. (2020) conducted another research to predict the potential invasion range of raccoons in Iran under the climate change effects using the ensemble model method. The results Illustrated that, under the climate change effects in Iran, raccoons could expand throughout a large portion of the country until 2050, thus managing this alien invader now would be more than necessary.

Given that limited studies have been conducted on this issue in Iran, we decided to rely on the studies that have been conducted on native and invasive raccoons in other parts of the world in order to gain as much information as we could perform one of the most comprehensive studies on this animal in Iran. There are many studies conducted on native and invasive raccoons around the globe, and they've used different methods and analyses (e.g., MaxEnt, home range evaluation, live‐trapping and surveying the trap locations, statistical analyses, ensemble methods, etc.) to evaluate environmental drivers affecting raccoon settlements and distribution (Baldwin et al., 2006; Barding & Nelson, 2008; Mori et al., 2015). The results of researches that are conducted using MaxEnt show that urban areas and their suburbs, the ratio of the coniferous forests, elevation, and temperature are the most important environmental drivers of raccoon settlement. It has been determined that global warming and increasing temperatures would have a massive effect on further expansions and could result in raccoon settlement in newer regions (Cunze et al., 2023; Duscher et al., 2018; Louppe et al., 2019; Mori et al., 2015). Other conducted methods such as home range evaluations, habitat selection patterns, live‐trapping, and hunting bag data; showed that edge of forests, wetlands, and waterbodies as well as hollow trees, trees with cavities, and distance to roads, are in the most decisive factors of raccoon establishment (Baldwin et al., 2006; Barding & Nelson, 2008; Fischer et al., 2016).

Given that previously conducted researches in Iran analyzed some parts of raccoon invasion in Guilan Province, we decided to survey all regions of the Guilan Province in which raccoon presence has been identified and recorded through confirmed reports of researchers, rangers, and environmental protection staff, during about 30 years. It is noteworthy that, there are no confirmed reports of raccoon presence in other parts of Iran (specifically northern provinces which have the valuable Hyrcanian forests).

**FIGURE 1:**
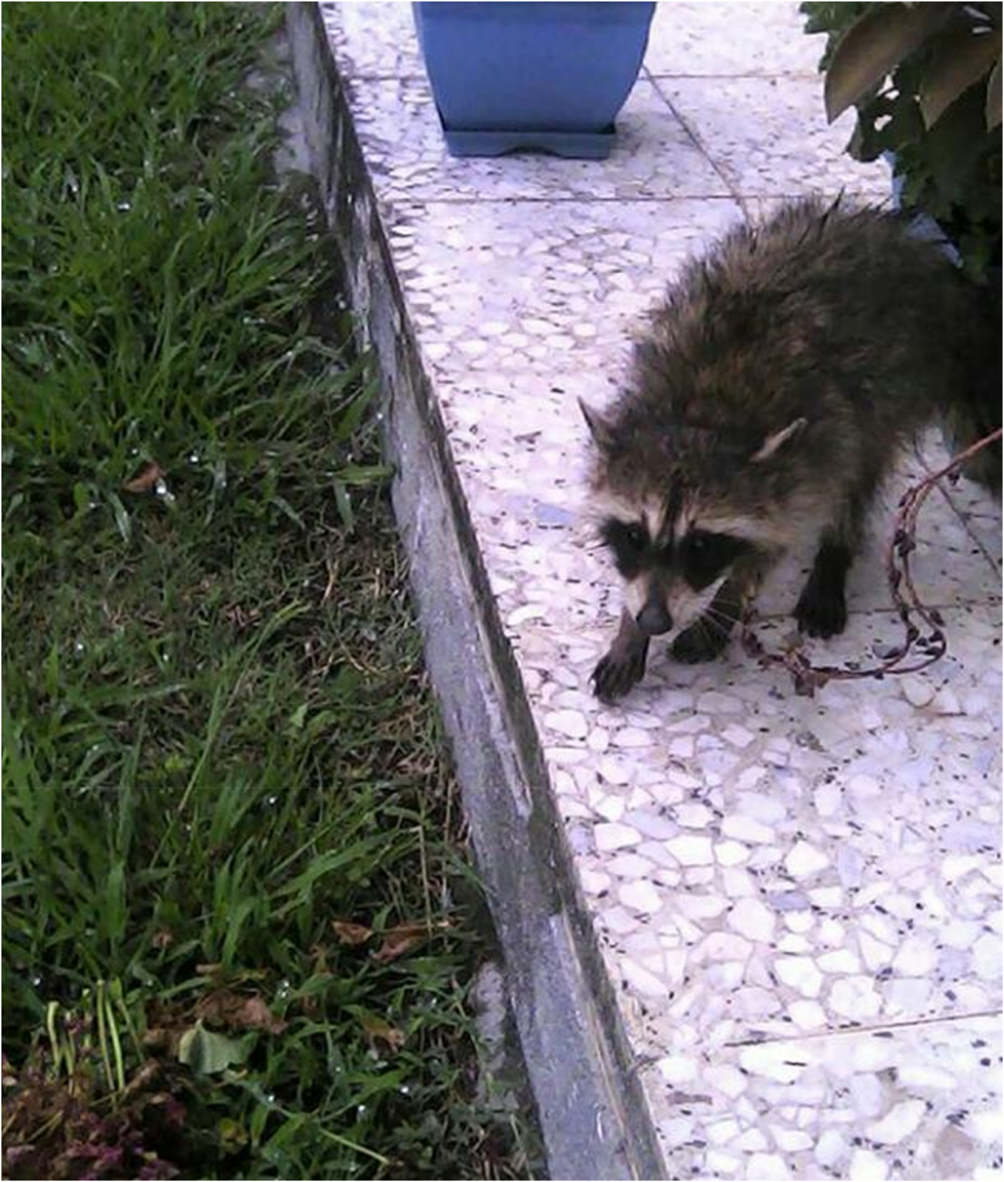
Raccoon presence in one of the houses in Rik village (Guilan Province). During the data recording process, many direct observations have been recorded in both natural and urban areas. This photograph represents the conflicts between raccoons as an invasive species and local people in the study region which cause much economic damages to these people.

In the present study, the habitat suitability of invasive raccoons in the Guilan Province of Iran was evaluated. Our goal was to determine the environmental factors of raccoon settlement in Iran and also compare these factors with other parts of the world. In our perspective, habitat suitability evaluation is a solid way to determine the status of the habitat regarding the establishment and expansion of this invasive species in order to control and reduce its negative effects (Figure 1).

## 2 MATERIALS AND METHODS

### 2.1 Study area

**FIGURE 2:**
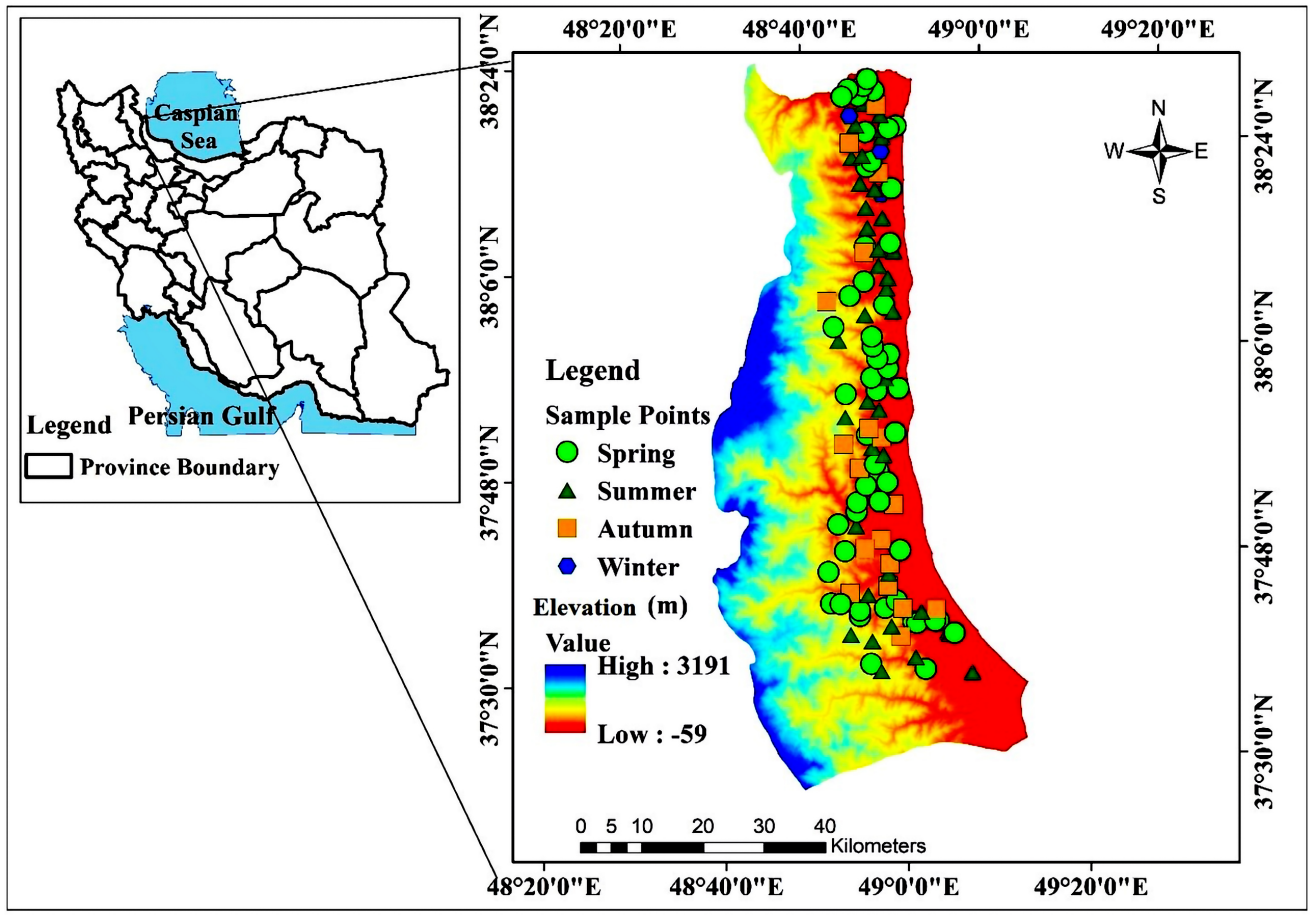
Geographical location of the study area and the recorded samples of Raccoon.

In this study, the western region of Guilan Province in the north of Iran was studied (Figure 2). This region is located in west of Guilan Province, from Rezvanshahr to Astara (coordinates 48° 55′ 10″ to 48° 19′ 98″ East longitude, and 37° 38′ 64″ to 38° 45′ 92” North latitude) and includes the cities of Rezvanshahr, Parehsar, Asalem, Talesh, Lavandevil, and Astara. Each city has a vast number of villages around it. The overall area of the study region is 332,612 hectares. The average elevation of the region is 938 meters, and the minimum and maximum elevations are −59 and 3191 meters above sea level, respectively (Figure 2).

### 2.2 Data record

Considering the vast area of the study region, we came up with a way by which we could record high quality with the most accurate data from all over the study region in each season of autumn 2019 to summer 2020. We divided the entire study region into sub‐regions based on the cities within them. Then again, we divided these sub‐regions into smaller areas. Afterward, we started collecting daily data from these smaller areas by surveying them on foot. Each of these smaller areas was surveyed twice a season. For example, we considered Talesh city as a sub‐region and divided it into villages and natural areas of the sub‐region (not only the villages but all of forests and natural areas); such as Rik village (and the forest surrounding), natural areas in within the smaller region based on the area, Gisoom beach (and the forest surrounding), Siahbil village and so on. The sampling process was done in both natural areas and rural areas as well. All of the research team were involved in the surveying process. We collected only presence data in each one of these small areas day by day, by surveying the natural areas, forests, mountains, pastures, open plains, villages, and nature around the villages (not specifically just nature adjacent to the village but the whole forest in the area), roads, rivers, and private properties. The data recording process started from 10 to 18 for daytime surveys and 20 to 4 for nights. We also asked the local people about this invasive species. We aimed to evaluate the knowledge of local people about the potential dangers of raccoons on wildlife and human health (which was very poor). By applying this method, we were able to collect data from all over the western Guilan in each season from east to west of the region.

We succeeded to collect data in forest, non‐forest (pastures, grasslands, agricultural lands, wetlands), urban, and rural areas. These data included direct (*n* = 73) and indirect (*n* = 41) observations such as feces, footprints, hunting traces, etc. (*n* = 114), which were recorded using GPS (Garmin, eTrex vista) and mounted to ArcGIS software (10.5 version) for creating maps and analysis. In direct observations, 42 cases were observed and recorded at night using two powerful flashlights for each researcher (one headlamp and one flashlight in hand), and 31 cases were recorded during the day. Of a total of 114 points, 54 points were recorded in spring (highest), 39 in summer, 18 in autumn, and 3 in winter (lowest) (which have been recorded around restaurants and in private properties with food sources available all around the season (houses with chickens and fruit trees)) (Figure 2). You can access the detailed coordinates of the presence points in supplementary materials.

### 2.3 Environmental variables and data analysis

One of the main goals of this study was to evaluate environmental drivers of raccoon, as an invasive species in Iran. According to the ecological and behavioral characteristics of invasive raccoons in our study area and considering other researches, eight environmental parameters were selected including; elevation, Normalized Difference Vegetation Index (NDVI), land use (with nine categories: 1: man‐made (urban areas), 2: low‐density forest, 3: High‐density forest, 4: water bodies, 5: medium density pasture, 6: agricultural land, 7: wetland, 8: High‐density pasture, and 9: medium‐density forest) distance to villages, distance to roads, distance to waterways, average temperature and rainfall (Bartoszewicz et al., 2008; Duscher et al., 2018; Fiderer et al., 2019; Gehrt, 2004). In addition, we added four more parameters including slope, aspect, geology, and soil type to evaluate their impact on the habitat suitability of invasive raccoons and gain as much as possible information about this species in order to control it. Each parameter was prepared through its specific satellite image, or maps obtained from official organizations and used in ArcGIS 10.5 software (Table 1).

**TABLE 1:** List of the environmental variables used for the analysis.

Variable	Source
Elevation	Aster sensor – Terra satellite
Slope	Aster sensor – Terra satellite
Aspect	Aster sensor – Terra satellite
Geology	Geological survey and mineral exploration of Iran
NDVI	Landsat 8 satellite
Land use	Landsat 8 satellite
Soil type	Natural resources and watershed management organization of Iran
Distance to village	Google Earth
Distance to road	OpenStreetMap
Distance to waterways	Aster sensor – Terra satellite
Average temperature	Iran meteorological organization
Rainfall	Iran meteorological organization

We have chosen to use Species Distribution Models (SDMs) (specifically the MaxEnt method) for data analysis. SDMs (also known as habitat modeling, ecological niche modeling, etc. (Elith & Graham, 2009)) are computational algorithms used for predicting the distribution of a given species across a geographical space using a variety of data (Austin, 2007). In this study, considering the features of the MaxEnt method such as; using presence‐only data (because of time and budget limits of this study), higher accuracy compared to other presence‐only methods (such as GARP) (Li & Wang, 2013; Phillips et al., 2006), higher model fitness to the study (species, data, area of the region, etc.) compared to other SDMs like the random forest, ensemble, artificial neural networks, etc. (Li & Wang, 2013), more robust results from other similar studies around the world with the same geographical and climatic conditions as this study which used MaxEnt method for habitat modeling (e.g., Duscher et al., 2018; Mori et al., 2015), vast area of the study region, we decided to use the MaxEnt method for habitat suitability analysis. Maximum Entropy method (MaxEnt) as a correlative niche model is a general‐purpose machine learning and frequently applied method for modeling species geographical distributions. The rationale of MaxEnt is to estimate a target probability distribution by finding the probability distribution of maximum entropy, subject to a set of constraints that represent the species distribution (Phillips et al., 2006).

We used the subsample algorithm of MaxEnt for habitat suitability analysis, and 70% of the data were set as training and 30% as testing data. Considering the most accuracy, the number of background points was set to 100,000 (AUC = 0.848), and given the vast area of the study region, the maximum iteration was set to 5000 and the model was repeated 50,000 times. In addition, for checking the validity of the model, we used the area under the curve (AUC) of the receiver operating characteristic (ROC) curve and according to that, if the value of the area under the curve (AUC) is between 0.7 to 0.8, 0.8 to 0.9, and more than 0.9, the model is considered good, very good and excellent, respectively (Pearson, 2007). For determining the validity of the parameters, the Jackknife method has been used. Examining the results of the approach obtained from the Jackknife diagram to investigate the importance of each parameter, by implementing the subsample algorithm shows that this curve represents the AUC value in three different modes. The first case represents when modeling is done by deleting the desired variable, that is, when the model is executed using all variables except the desired variable. In the second case, modeling is done with only one variable. In this test, environmental variables show their greatest effect when used alone to implement the model and provide the most useful information. The third case also indicates when the modeling is performed in the presence of all variables (Phillips & Dudík, 2008; Phillips et al., 2006). Also, Analysis and the impact of each parameter on the suitability of the habitat is very crucial. Considering that Maxent is a machine learning method, the probability that it refers to each location is proportional to the exponential view of the selected parameters. Therefore, it is possible to construct response curves to describe the effect of selected parameters for probability used in that location (Eastman, 2015). Furthermore, since all three northern provinces of Guilan, Mazandaran, and Golestan share the same ecosystem known as Hyrcanian forests, which contains a very rich diversity in fauna and flora, there is the probability of invasive raccoon expansion toward two other northern provinces of Iran. Thus, we extended the habitat suitability modeling for all regions of three provinces using the data obtained in the study region of this research.

## 3 RESULTS

**FIGURE 3:**
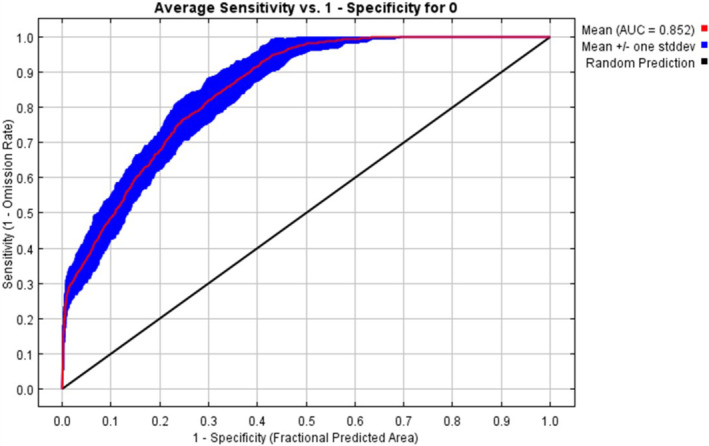
Validity curves of the habitat suitability model based on the value of AUC.

**FIGURE 4:**
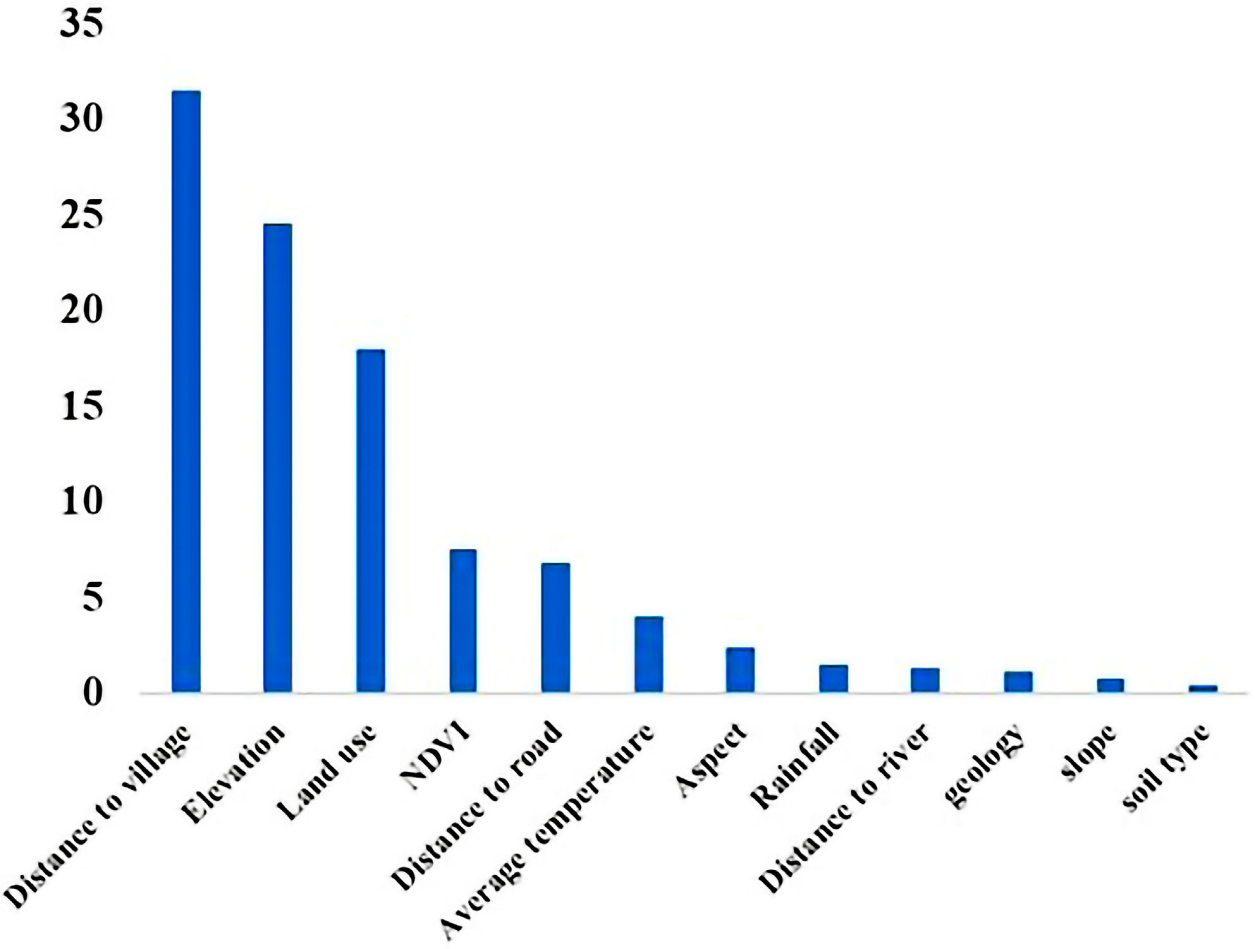
Contribution percent of each parameter to the habitat suitability of raccoons in the western region of Guilan Province.

The correlation between variables in all cases was less than 0.85. Thus, none of the variables were deleted. The amount of AUC obtained was 0.852. The validity value for training and testing data was 0.901 and 0.850 respectively, indicating that the discrimination power of the model is considered robust and can accurately identify different areas of habitat suitability (Figure 3). The percentage of the relative contribution of each variable in the distribution of raccoons in the western region of Guilan Province, using the subsample algorithm in the model, shows that the most effective parameters on habitat suitability are distance to village (34.5%), elevation (24.2%), and land use (15.9%) and least effective parameters are slope (1%), soil type (1%), and distance to waterway (1.5%) (Figure 4).

**FIGURE 5:**
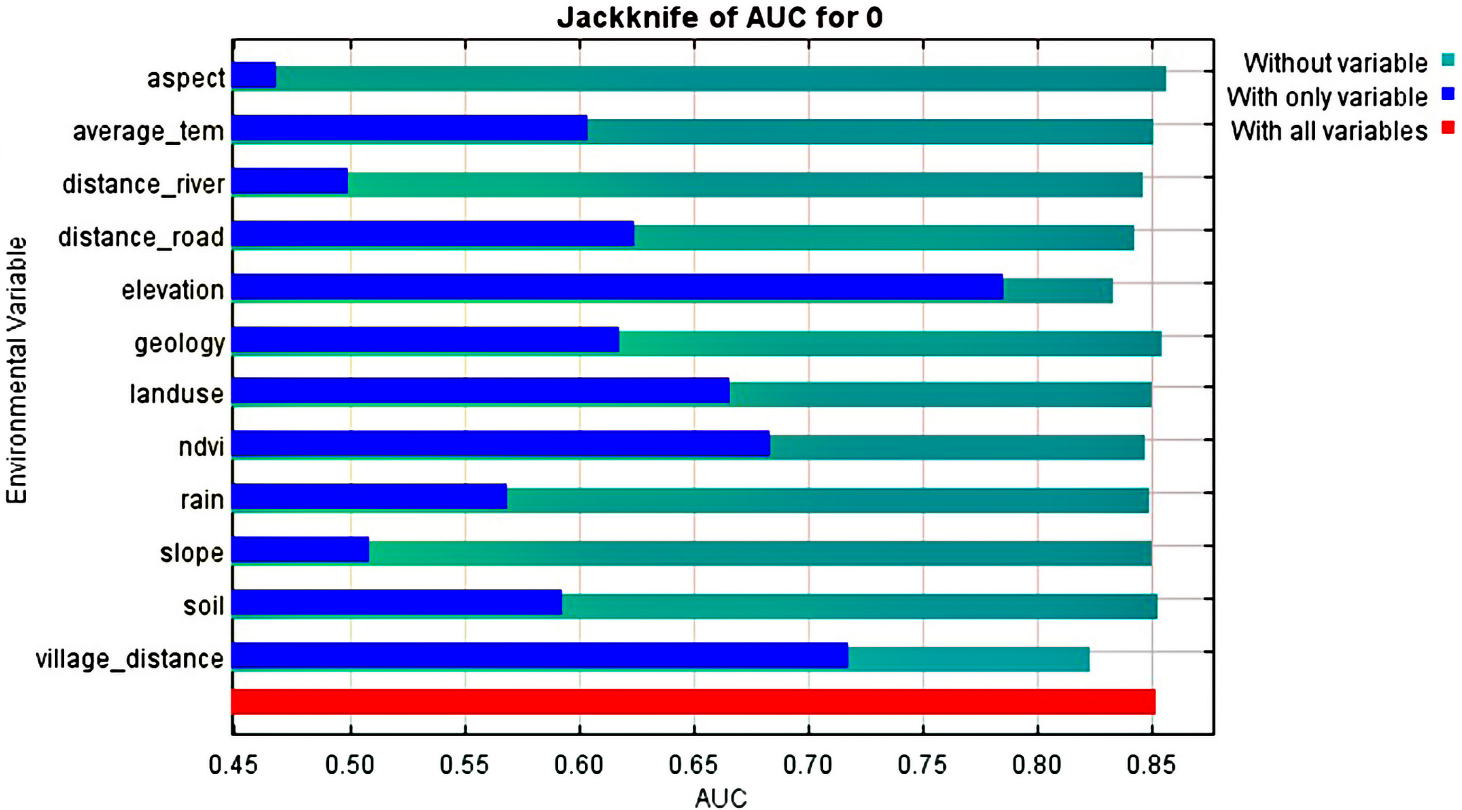
Results of the Jackknife test to investigate the contribution of different parameters in habitat suitability of raccoons in the west of Guilan Province.

According to obtained results from the Jackknife method (Figure 5), aspect, geology, and soil type are variables that when removed, the most decrease in AUC has been observed. Also, in case of implementation of this test with each variable alone, the variables of elevation, distance to village, and NDVI have produced the highest AUC.

The results of response curves of environmental parameters are given as follows. With increasing elevation up to 300 meters, the suitability increases exponentially. However, elevations higher than 300 meters (up to 1700 meters and more) show a sudden decreasing rate of suitability that ultimately shows a steady rate in elevations higher than 1700 meters. The response curve of slope shows that increasing slope up to 2.22%, increases the suitability and gives the highest suitability which, after that, with a higher slope, the suitability decreases rapidly. Results showed that west and north aspects have the highest suitability and the lowest amounts belong to southeast, southwest, and northeast directions. In case of NDVI, the response curve shows that up to 0.2, there is a constant suitability, and higher than 0.2 up to 0.55, the suitability increases and reaches the threshold at which after that rate, suitability drops significantly. For distance to village, up to about 100 meters to villages, there is the most habitat suitability, and with increasing this distance to 300 meters, the desirability has decreased significantly and then the suitability decreases slowly. The rainfall response curve showed that with increasing up to 900 mm, the suitability is almost constant and high, but higher amounts of rainfall decrease the suitability significantly. For average temperature, results showed that at 16.6°C, there is the suitability threshold, but lower and higher temperatures will decrease the suitability significantly.

**FIGURE 6:**
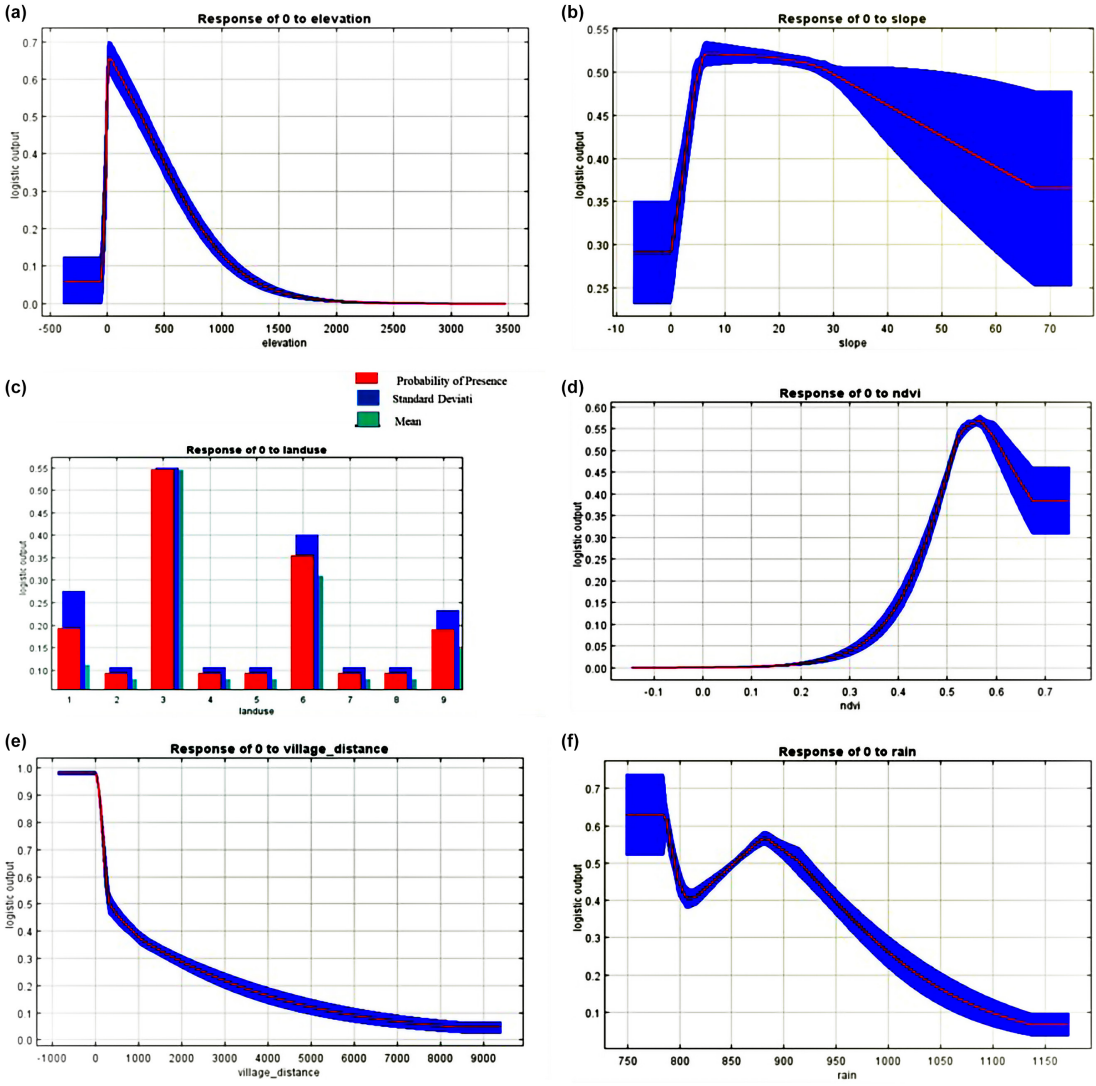
Response curves of environmental parameters; (a): Elevation, (b): Slope, (c): Land use (1: Man‐made (urban areas), 2: Low‐density forest, 3: High‐density forest, 4: Water bodies, 5: Medium‐density pasture, 6: Agricultural land, 7: Wetland, 8: High‐density pasture, and 9: Medium density forest), (d): NDVI, (e): Distance to village, (f): Rainfall, (g): Average temperature.

**FIGURE 7:**
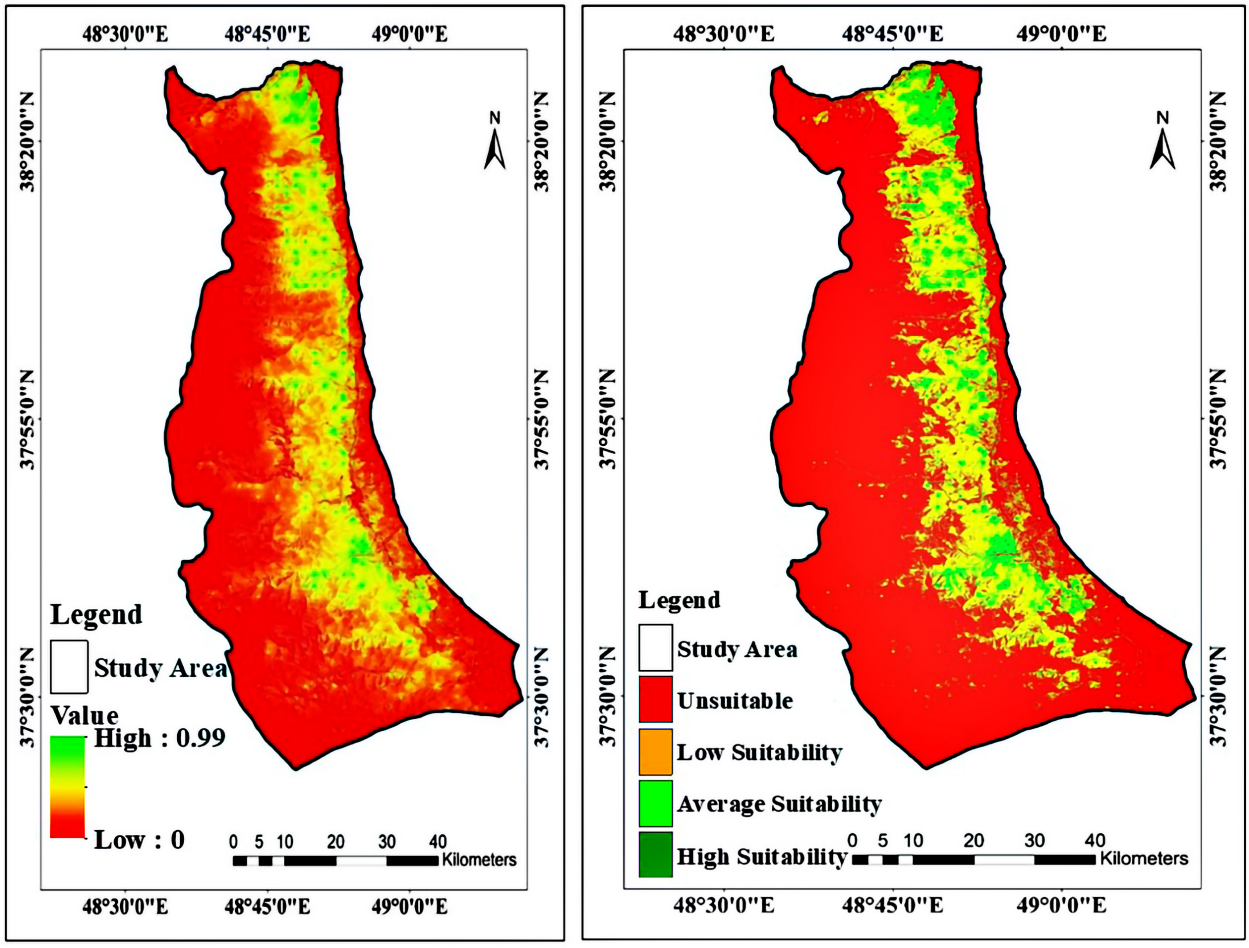
Habitat suitability map (left) and classified habitat suitability map (right).

For alien raccoon, 300 m elevation above sea level, 2.22% slope, north and west directions, dense forests, agricultural lands, medium‐density forests, followed by urban or rural areas, 0.55 rate of NDVI, distance to village up to 100 m, areas with annual rainfall of 780 mm, the average annual temperature of 16.6°C is the most desirable areas in western regions of Guilan Province (Figure 6). By applying different analyses, the final habitat suitability map and the percent of different suitability levels of habitat regions were obtained (Figure 7, Table 2).

**TABLE 2:** The area and percentage of different habitat suitability classes.

Classification	Percentage	Area (hectares)
Unsuitable	68.36	226,646.1
Low suitability	24.87	82,477.23
Average suitability	6.14	20,738.99
High suitability	0.60	2002.57

**FIGURE 8:**
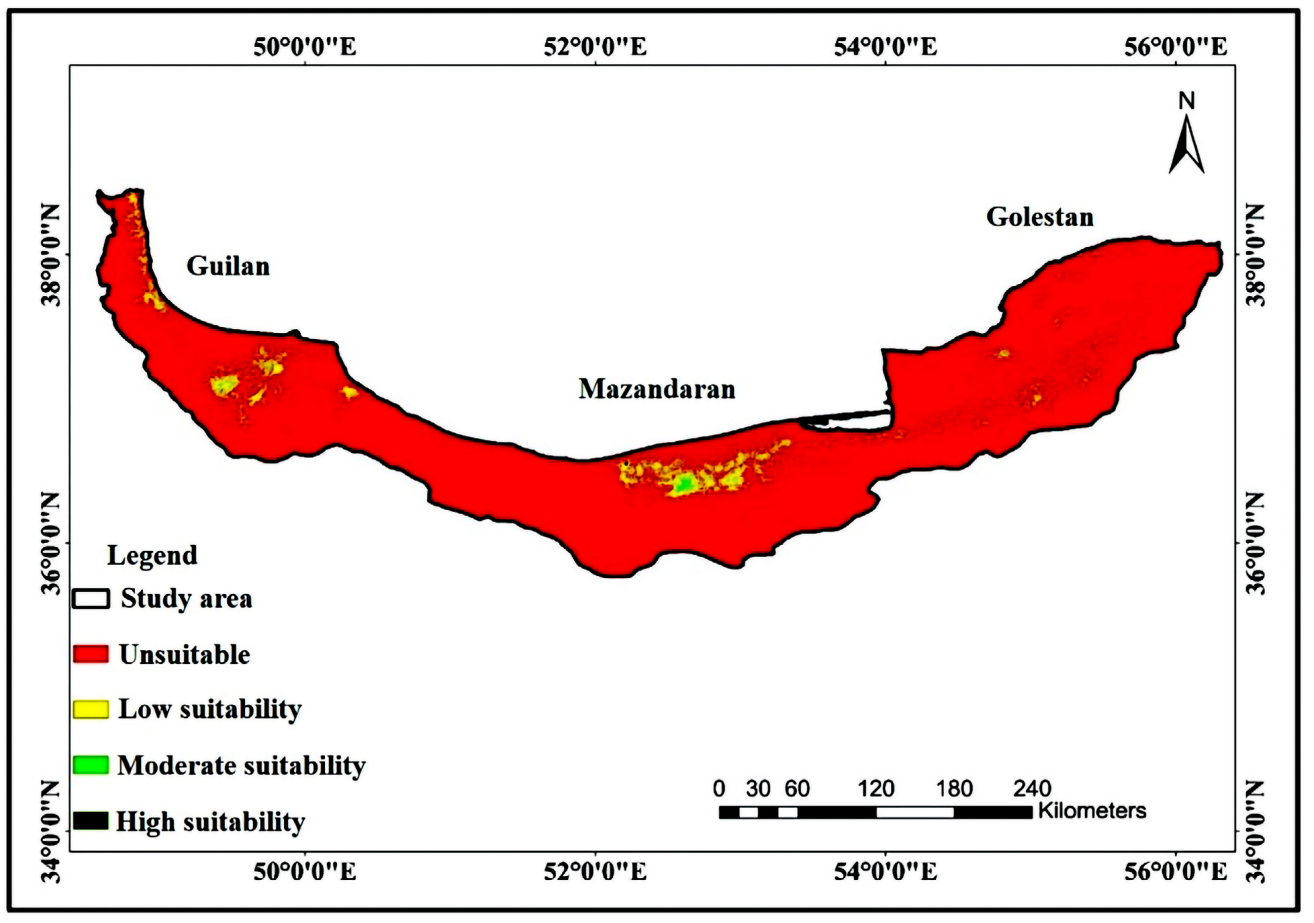
Integrated habitat suitability map of all three northern provinces of Iran.

Also, Figure 8 shows the habitat suitability map for all three northern provinces of Iran, based on the expansion of invasive raccoons. As seen, all three northern provinces of Iran have suitable parts but most of the areas are unsuitable or have a low suitability for raccoon. But as mentioned before, this analysis shows a potential threat of an opportunistic species with a wide range niche. It is also noteworthy that by taking a glance at Figure 8, it is easy to see a narrow corridor of areas with low and moderate suitability that could facilitate this expansion.

## 4 DISCUSSION

The aim of this research was to investigate the habitat suitability of raccoons in the western region of Guilan Province in order to achieve the applied information for appropriate management of this invasive species. We started this study with hypotheses such as the presence of raccoons depends on waterways, villages, topographic variables, and vegetation. The results obtained proved all the hypotheses. In other words, we wanted to compare the distribution pattern of invasive raccoons and factors affecting it in Iran with other regions of the world in which invasive raccoons existed or still existing. The obtained results in the present study showed that raccoon ecology in western Guilan does not have many differences compared to the other parts of the world, and this invader prefers approximately similar habitats to settle (Bartoszewicz et al., 2008; Beasley et al., 2007; Duscher et al., 2018; Fiderer et al., 2019; Fischer et al., 2016; Henner et al., 2004; Heske & Ahlers, 2016; Ikeda et al., 2004). Given that the validity values of training and testing data are 0.901 and 0.850 respectively, it is safe to say that the performance of the model is at an excellent level, thus the accuracy of the model is well proved.

Even though only 0.60% of the study area is completely suitable for raccoons (plus about 30% of relatively suitable regions), it does not mean that only these small areas should be managed. Instead, it means that all of the favorable factors that cause the most suitability can be seen altogether in limited places at the study region. But the important point is that being a generalist and opportunist species with a wide niche allows raccoons to settle in areas in which there are only 2 or 3 favorable factors unless the existence of unsuitable lethal factors (i.e., very low temperatures (Farashi et al., 2016; Mori et al., 2015)). So that, there are many reports and documents of different damages to raccoons (agricultural, kinds of conflicts, disease, etc.) in our study areas over the years. Considering that the raccoon invasion in Iran started in 1996 (according to Ziaei, 1996), this result is probably due to an ongoing invasion that has not been finished yet. Therefore, raccoons are not probably in equilibrium with the environmental conditions of Guilan Province, which could challenge the applications of SDMs. But as Kochmann et al. (2021) and also Cunze et al. (2023), using MaxEnt model, indicated that further invasion of raccoons in Europe is possible, thus this assumption could probably happen for invasive raccoons in Iran, too.

The most presence points were recorded during spring and in areas such as forests, agricultural lands, around roads, villages, and waterways. Selecting these areas is because of available food and water sources as well as abundant shelters (Bartoszewicz et al., 2008; Duscher et al., 2018; Fischer et al., 2016; Ikeda et al., 2004; Khosravifard et al., 2020; Osaki et al., 2019; Schell et al., 2021). Considering that only 3 points were recorded during winter (which were recorded near the restaurants and food courts), it can be concluded that raccoons in Iran, as well as other parts of the world, have winter denning (hibernation) and cannot tolerate cold temperatures of winter and therefore, constant monitoring in summer and spring seasons would be beneficial to gain more information of this animal and perform better management strategies (Bartoszewicz et al., 2008; Folk Jr et al., 1968; Mazzamuto et al., 2020).

By looking at the presence points map (Figure 2), it is clear to see that all of the presence points were recorded on a gradient of areas and other parts of the region do not have any points. There are two main reasons for this phenomenon: (i) the land uses, such as pastures and grasslands with moderate vegetation and low‐density forests which are not suitable for raccoon settlement, and (ii) 1500 to 3191 meters of elevation, which is not favorable for raccoons. Therefore, these areas will not be selected by raccoons due to low annual temperatures, lower vegetation density, and weaker biodiversity compared to lower elevations. These high elevations are currently functioning as a barrier to raccoons' expansion to western parts of the country. Due to the fact that the average temperature of the region has a significant effect on the presence of the species in that region (Duscher et al., 2018; Farashi et al., 2016; Fiderer et al., 2019; Mori et al., 2015), so, with increasing elevation, the average temperature and the number of warm days decreases. Therefore, raccoons do not prefer such high elevations. According to studies, although invasive raccoons have been seen in colder parts of the world, they often prefer areas with relatively high temperatures throughout the year. In other cold regions, invasive raccoons settle only when their most basic habitat needs (such as shelter and food) are available (Duscher et al., 2018; Mori et al., 2015). On the other hand, the reduction of animal and plant diversity at very high elevations is another reason for this phenomenon. Duscher et al. (2018) also modeled the habitat suitability of invasive raccoons in Austria and concluded that high elevation has a severely negative effect on the presence of the species. They attributed this to the decrease in the average temperature of the region and the number of warm days of the year, as well as because of snow cover on most days of the year.

Also, the results showed that, by increasing the amount of normalized vegetation difference index (NDVI) to 0.55, the habitat suitability rate of invasive raccoons increases, and after that, the degree of suitability decreases. The results of the response curve in land use also showed that the raccoon prefers denser and medium‐density forest areas. It seems that this species selects such areas that have suitable vegetation. Because raccoons often choose forest areas (especially deciduous forests) for important biotic needs, such as reproduction and shelter, and often do not prefer open areas. Duscher et al. (2018) also concluded that the probability of the species presence in coniferous forests of Austria initially increases by 40% and then decreases. They attributed this to the greater preference for deciduous forests over coniferous forests and also stated that because coniferous forests are denser at higher elevations (which is undesirable for raccoons), the lower elevations with less coniferous forest density will be more desirable.

Based on the obtained results of this study, the presence of the species is higher in areas close to the villages and human settlements. Therefore, it can be concluded that the presence of the species is highly dependent on rural areas and food sources near and inside the villages. This is due to the existence of numerous and varied food sources such as garbage, fruit trees, and birds such as chickens and ducks that are maintained by the villagers. During interviews with people living in the villages, it was found that raccoons use these food sources frequently, especially fruits, eggs, and ducks, which is one of the most important reasons for their dissatisfaction with the presence of this invader species in the region. The results of Duscher et al. (2017) also showed that there is a positive relationship between human settlements and raccoon presence. So that in residential areas, the presence of the species increases significantly. This is also consistent with the results of Ikeda et al. (2004). In these two studies, the most important reason for the selection of residential areas by raccoons is access to diverse and permanent food sources. Other studies support this result as well (Fiderer et al., 2019; Heske & Ahlers, 2016; Hohmann & Bartussek, 2005).

So, what are the potential dangers of raccoon's consistent presence in this region? Considering that this invasive species is placed in mid‐levels of the food web, it could have many direct and indirect negative effects on a wide range of animals including hunters and preys (Gehrt, 2003; Kaufmann, 1982). These effects could damage the balance of the hunter–prey relations, thus causing damage to native fauna and flora (Ikeda et al., 2004). By hunting the small preys, raccoons could constrain the food security of bigger hunters such as leopard, red fox, jungle cat, etc., and considering the critical state of these species in local ecosystems; it could result in biodiversity disasters, thus appropriate management actions to remove this masked invader would be more than necessary (Bartoszewicz et al., 2008; Duscher et al., 2018; Gebhardt, 1996). Besides the local mammals, local birds are also in danger of raccoons, such as direct hunting or nest hunting. As seen before in Japan, invasive raccoons invade the nests of owls (which mostly are hollow trees), discharge the nest, and feed on owl eggs, thus endangering the survival and breeding and ultimately cause habitat dislocation of these local birds, which could end up in local extinction (Ikeda et al., 2004). Apart from direct effects, an increase in the population of raccoons increases the risk of disease transition (rabies, raccoon roundworm, distemper), which endangers both human and ecosystem health (Beltrán‐Beck et al., 2012; Kornacka et al., 2018; Rentería‐Solís et al., 2018). Furthermore, during the data record, we did many interviews with medical staff all over the study region, and no evidence of rabies transition was found, but that does not mean that local people are safe. On the contrary, since the local people have very limited information about this invasive animal and its possible harm to human health, the threat of getting rabies from raccoons is very serious and it could turn into a real danger to public health (Arjo et al., 2005).

As seen before in Canada (Larivière, 2004), before the 1960s and 1970s, raccoon was only limited to southern parts of Canada, but after the 1960s and 1970s it expanded northward due to agricultural development and global warming. The same thread is expected for raccoon expansion in Iran, too. With global warming and warmer temperatures, high elevated areas, which nowadays are unsuitable for expansion due to cold annual temperatures, will get warmer. This increase in average temperature will cause flora changes in higher elevations (Kc & Ghimire, 2015) and plants that cannot survive in lower temperatures would reach higher elevations. With suitable flora, the local fauna will reach those elevations, too. Thus the conditions for raccoon settlement will be more favorable; and probably, the Alborz mountains which play a role of natural barrier for raccoon expansion westward and southward will become a corridor with low suitability and that could result in raccoon settlement in Zagros mountains which is an important habitat for valuable brant's oak (*Quercus brantii*). With raccoons' settlement in the Zagros ecosystem, it would disrupt the ecosystem balance by hunting the Caucasian squirrel (*Sciurus anomalus*) which is a keystone species in the Zagros region (Karami et al., 2016). Khosravifard (2015) and Khosravifard et al. (2020) also investigated the potential expansion of invasive raccoons in Iran and results showed that raccoons could expand to western and even southwestern parts of the country by 2060s.

Apart from the western regions of Iran, northern provinces are in danger too. As seen in Figure 8, as the western region of Guilan Province, the eastern regions also have some suitable and moderately suitable areas, which would be the first probable station in case of potential eastward expansion of raccoons. Mazandaran Province, which has significant amounts of suitable habitats would be the second station of this potential expansion. Even though the absence of suitable and even merely suitable areas between Guilan and Mazandaran provinces would be a key component in stopping raccoon expansion from Guilan to Mazandaran, but being a generalist invasive species with wide niche and opportunistic behaviors would help raccoons to tolerate the unsuitable areas with one or two favorable components to reach suitable regions of Mazandaran Province. Furthermore, by taking a glance at Figure 7, there is a corridor of suitable and moderately suitable areas between the Mazandaran and Golestan Provinces which could play an important role in the potential expansion of raccoons from Mazandaran to Golestan and considering the numerous suitable and moderately suitable areas of Golestan Province, it is safe to conclude that this invasive species would expand rapidly throughout Golestan province, too. This potential expansion to all three northern Provinces of Iran would be a great danger to Hyrcanian ecosystems which is a valuable and unique ecosystem with many valuable local and endemic animal and plant species, some of which are endangered and critically endangered.

Finally, we came up with solutions for managing this invasive species: 1. Habitat improvement with an approach of increase in population of local hunters that could play a role as natural enemies 2. Order to rangers and hunters for complete eradication. This solution has worked before in other parts of the world such as Japan and some parts of Canada (Golumbia et al., 2000; Ikeda et al., 2004). Also, in a more precise and accurate way, doing a complete eradication in the way Mazzamuto et al. (2020) did in Lombardy, Italy, if implemented with the same precision and accuracy, would be a great help for complete clearing the region of this invasive mammal and also engaging stakeholders in the process of these projects would be very much beneficial. Therefore, a strong monitoring program and gaining more data about raccoons would be more than necessary to perform this management strategy 3. Presenting rewards in exchange for the animal carcass in order to motivate local hunters; considering that this solution could lead to some kind of secondary business for freeloaders (not killing all raccoons to maintain the population for more hunts and more rewards) this procedure requires constant and direct monitoring of stakeholders and rangers to prevent these kinds of businesses 4. Given the poor knowledge of local people about raccoon and its potential dangers, informing people through seminars, classes, etc. would be essential. And to future researchers in this field, we recommend investigating the food diet of raccoons, performing strong monitoring research specifically in spring and summer seasons (which raccoon presence is better), ecological impacts on hunter–prey relations and ecosystem balance in the study region, which could cause in more robust information thus better management of this invasive species.

Given that this research was done on personal expense and for a Master's degree thesis, there were some limitations regarding budget and time as well as transportation, causing us to ignore expensive (in time and budget) sampling methods such as collecting absence points, using camera traps or live‐trapping methods. Certainly, more supported researches in future would be very beneficial in managing invasive raccoons in Iran.

Considering that some of the studies in this field are focused on large scales such as countries or even continents nowadays, the scale of this study may seem more undersized. However, compared to studies conducted in Iran, and considering the mentioned limitations regarding budget and time, our study has the biggest sampling area scale in Iran. Other studies in Iran have sampled only some parts of raccoon presence regions in Guilan Province, while we collected data from all parts of Guilan Province where raccoon presence has been identified through verified reports of environmental protection staff and rangers. This is the biggest sampling scale study that has ever been performed in Guilan Province.

## AUTHOR CONTRIBUTIONS

**Amin Hekmat:** Conceptualization (lead); data curation (lead); formal analysis (lead); investigation (lead); methodology (lead); project administration (equal); resources (lead); software (lead); validation (lead); visualization (lead); writing – original draft (lead); writing – review and editing (lead). **Saeid Naderi:** Conceptualization (supporting); data curation (supporting); formal analysis (supporting); investigation (supporting); methodology (supporting); project administration (equal); supervision (lead); validation (supporting); visualization (supporting); writing – original draft (supporting); writing – review and editing (supporting). **Wahid Zamani:** Software (equal); supervision (supporting); validation (equal); writing – original draft (supporting); writing – review and editing (supporting).

## CONFLICT OF INTEREST STATEMENT

We have no conflicts of interest to disclose.

## Supporting information


**Table S1.** Coordinates of raccoon presence points, recorded during the data record process.

## Data Availability

Data about the habitat suitability maps of this study are available from the Figshare digital repository (https://figshare.com/s/cd4c0c2cd7b8bb661024).
