# Supplementary material for: Masked invader in Iran! Habitat suitability analysis for invasive raccoon (Procyon lotor) in the west of Guilan Province
Source: Ecol Evol. 2024 Aug 6;14(8):e70090. doi: 10.1002/ece3.70090 (PMC11303449; doi:10.1002/ece3.70090)
Supplement: Supplementary file 1 — Table S1. Coordinates of raccoon presence points, recorded during the data record process. [file ECE3-14-e70090-s001.docx]

**Supplementary materials**

Table 1 shows the coordinates of raccoon presence points recorded during the data record process.

Table 1. coordinates of raccoon presence points, recorded during the data record process

| Row | Longitude | Latitude |
| --- | --- | --- |
| 1 | 49/01689911 | 37/65510178 |
| 2 | 48/97340012 | 37/64970016 |
| 3 | 48/97060013 | 37/65000153 |
| 4 | 48/85749817 | 38/37139893 |
| 5 | 49/03530121 | 37/64039993 |
| 6 | 49/03960037 | 37/63809967 |
| 7 | 49/04790115 | 37/63899994 |
| 8 | 49/00090027 | 37/58280182 |
| 9 | 48/97740173 | 37/64849854 |
| 10 | 49/01089859 | 37/65409851 |
| 11 | 48/87139893 | 37/65119934 |
| 12 | 48/87080002 | 37/65969849 |
| 13 | 49/08670044 | 37/58430099 |
| 14 | 48/89799881 | 37/61700058 |
| 15 | 48/94929886 | 37/62770081 |
| 16 | 48/90019989 | 37/58319855 |
| 17 | 48/91970062 | 37/57440186 |
| 18 | 48/85760117 | 37/62340164 |
| 19 | 48/98040009 | 37/59939957 |
| 20 | 48/91619873 | 37/66650009 |
| 21 | 49/00960159 | 37/67240143 |
| 22 | 48/98339844 | 37/66630173 |
| 23 | 48/93629837 | 37/67890167 |
| 24 | 48/94850159 | 37/66910172 |
| 25 | 48/93059921 | 37/6413002 |
| 26 | 48/9178009 | 37/69950104 |
| 27 | 48/8830986 | 37/68399811 |
| 28 | 48/84960175 | 37/68439865 |
| 29 | 48/81639862 | 37/66609955 |
| 30 | 48/83430099 | 37/66669846 |
| 31 | 48/91699982 | 37/71820068 |
| 32 | 48/9178009 | 37/73270035 |
| 33 | 48/83359909 | 37/74409866 |
| 34 | 48/80690002 | 37/71170044 |
| 35 | 48/89799881 | 37/76610184 |
| 36 | 48/93460083 | 37/75289917 |
| 37 | 48/86989975 | 37/75139999 |
| 38 | 48/84960175 | 37/78310013 |
| 39 | 48/8166008 | 37/78229904 |
| 40 | 48/84859848 | 37/80329895 |
| 41 | 48/91529846 | 37/81919861 |
| 42 | 48/84809875 | 37/81700134 |
| 43 | 48/88280106 | 37/84909821 |
| 44 | 48/88230133 | 37/8655014 |
| 45 | 48/86169815 | 37/84270096 |
| 46 | 48/84640121 | 37/86729813 |
| 47 | 48/87480164 | 37/875 |
| 48 | 48/90050125 | 37/85010147 |
| 49 | 48/86640167 | 37/89989853 |
| 50 | 48/88850021 | 37/89039993 |
| 51 | 48/81259918 | 37/90029907 |
| 52 | 48/85540009 | 37/91669846 |
| 53 | 48/88169861 | 37/91569901 |
| 54 | 48/90679932 | 37/92399979 |
| 55 | 48/85749817 | 37/92660141 |
| 56 | 48/88940048 | 37/82170105 |
| 57 | 48/87319946 | 37/95500183 |
| 58 | 48/85079956 | 37/96640015 |
| 59 | 48/81190109 | 37/93989944 |
| 60 | 48/80939865 | 37/97359848 |
| 61 | 48/90579987 | 37/98920059 |
| 62 | 48/86629868 | 37/98300171 |
| 63 | 48/87990189 | 38/00189972 |
| 64 | 48/85419846 | 38/00099945 |
| 65 | 48/88359833 | 38/01689911 |
| 66 | 48/88299942 | 38/03770065 |
| 67 | 48/86190033 | 38/02930069 |
| 68 | 48/85150146 | 38/04660034 |
| 69 | 48/78659821 | 38/05089951 |
| 70 | 48/84769821 | 38/06079865 |
| 71 | 48/83119965 | 38/09209824 |
| 72 | 48/86589813 | 38/10919952 |
| 73 | 48/8832016 | 38/10020065 |
| 74 | 48/86729813 | 38/13330078 |
| 75 | 48/82479858 | 38/14049911 |
| 76 | 48/84840012 | 38/16569901 |
| 77 | 48/86759949 | 38/14929962 |
| 78 | 48/87400055 | 38/18909836 |
| 79 | 48/82040024 | 38/19210052 |
| 80 | 48/81890106 | 38/18370056 |
| 81 | 48/86610031 | 38/19979858 |
| 82 | 48/82160187 | 38/21960068 |
| 83 | 48/84500122 | 38/18899918 |
| 84 | 48/84769821 | 38/23609924 |
| 85 | 48/81539917 | 38/24909973 |
| 86 | 48/84069824 | 38/27059937 |
| 87 | 48/82839966 | 38/27640152 |
| 88 | 48/80059814 | 38/28340149 |
| 89 | 48/85990143 | 38/27980042 |
| 90 | 48/83319855 | 38/30099869 |
| 91 | 48/81010056 | 38/30820084 |
| 92 | 48/81850052 | 38/31620026 |
| 93 | 48/78070068 | 38/31949997 |
| 94 | 48/79980087 | 38/32429886 |
| 95 | 48/83340073 | 38/33300018 |
| 96 | 48/83330154 | 38/35430145 |
| 97 | 48/80239868 | 38/35889816 |
| 98 | 48/83209991 | 38/36510086 |
| 99 | 48/84460068 | 38/36669922 |
| 100 | 48/7743988 | 38/3409996 |
| 101 | 48/8260994 | 38/38570023 |
| 102 | 48/78219986 | 38/36790085 |
| 103 | 48/76959991 | 38/38029861 |
| 104 | 48/79320145 | 38/40029907 |
| 105 | 48/81719971 | 38/3993988 |
| 106 | 48/81060028 | 38/42050171 |
| 107 | 48/7826004 | 38/41040039 |
| 108 | 48/79079819 | 38/42570114 |
| 109 | 48/76269913 | 38/41870117 |
| 110 | 48/75279999 | 38/4070015 |
| 111 | 48/79710007 | 38/43610001 |
| 112 | 48/80039978 | 38/11740112 |
| 113 | 48/75849915 | 38/10620117 |
| 114 | 48/77610016 | 38/06959915 |
